# Association between fasting Triglyceride levels and the Prevalence of Asymptomatic Intracranial Arterial Stenosis in a Chinese Community-based Study

**DOI:** 10.1038/s41598-018-24157-w

**Published:** 2018-04-10

**Authors:** Jianwei Wu, Yu Wang, Anxin Wang, Jian Xie, Xingquan Zhao

**Affiliations:** 1 Department of Neurology, Beijing Tiantan Hospital, Capital Medical University, Beijing, 100050 China; 2 China National Clinical Research Center for Neurological Diseases, Beijing, 100050 China; 3 Center of Stroke, Beijing Institute for Brain Disorders, Beijing, 100050 China; 4 Center of Brain Tumor, Beijing Institute for Brain Disorders, Beijing, 100050 China; 5 Beijing Key Laboratory of Translational Medicine for Cerebrovascular Disease, Beijing, 100050 China; 6 Beijing Key Laboratory of Brain Tumor, Beijing, 100050 China; 7 Department of Epidemiology and Health Statistics, School of Public Health, Capital Medical University, Beijing, 100050 China; 8 Department of Neurosurgery, Beijing Tiantan Hospital, Capital Medical University, Beijing, 100050 China

**Keywords:** Cerebrovascular disorders, Risk factors

## Abstract

The aim of this study was to assess the association between fasting triglyceride (FTG) levels and the prevalence of asymptomatic intracranial arterial stenosis (ICAS). The Asymptomatic Polyvascular Abnormalities Community (APAC) study is a sub-population of the Kailuan study which targeting on the epidemiology of asymptomatic polyvascular abnormalities in Chinese adults. A total number of 5345 participants, aged ≥40, and without history of stroke, transient ischemic attack, and coronary heart disease were enrolled in this study. Transcranial Doppler Ultrasonography was performed for the detection of ICAS presence. Out of 5345 participants, 698 subjects diagnosed ICAS (13.1%). In univariate analysis, the association between fasting TG (FTG) levels and asymptomatic ICAS didn’t reach statistical significance (OR: 0.99, 95% CI: 0.89–1.09; P = 0.79), the same conclusion was reached in multivariate analysis, after adjustment for age, sex (OR: 1.05, 95% CI: 0.95–1.17) and age, sex, current smoking status, hypertension, diabetes, body mass index, estimated glomerular filtration rate, total cholesterol, high-density lipoprotein cholesterol and low-density lipoprotein cholesterol (OR: 0.95, 95% CI: 0.84–1.06), respectively. FTG failed to show any statistical significance on ICAS presence in the APAC study, but the TG actually plays an important role in the progression of atherosclerosis as a biomarker.

## Introduction

Intracranial arterial stenosis (ICAS) is the result of atherosclerosis that affects large intracranial arteries^1^, which is a major cause of ischemic stroke worldwide^2^, especially in Asia^3^. It has been estimated that 33% to 84% of Asian ischemic stroke patients suffered from ICAS, both symptomatic and asymptomatic^4^. Since atherosclerotic lesions grow beneath the surface for several years until they become symptomatic, the early intervening treatment for asymptomatic phase, as a predictor for future stroke, is necessary and more effective.

Hyperlipidemia is significantly associated with the severity of ICAS^5^. Low-density lipoprotein cholesterol (LDL-C) and total cholesterol (TC) are well-established risk factors for atherosclerotic cardiovascular diseases (ASCVD, including ischemic stroke, myocardial infarction, ischemic heart disease). A number of large-scale epidemiological studies have shown that elevated triglyceride (TG) levels are substantial residual cardiovascular risk, even in patients received significant LDL-C lowering therapy with statins^6–8^.

Hypertriglyceridemia, defined as a TG level of ≥150 mg/dL (≥1.7 mmol/L), is brought to the forefront, act as a mediator of atherosclerosis with the growing epidemic of obesity, metabolic syndrome, type-2 diabetes mellitus^9^. Controversial results observed in studies between TG and ASCVD. Several studies demonstrated a univariate association between them, while after adjustment for other lipid parameters, particularly high-density lipoprotein cholesterol (HDL-C), the association is attenuated or non-significant^10–13^. But, it is worth noting that the reduction of TG pharmacologically by fibrate^14^, niacin^15^ or omega-3 fatty acids^16^ can lower the risk of ASCVD, acting as an effective preventive strategy. While recognizing this possibility, we therefore conducted this epidemiologic and observational study to investigate an association between FTG levels and asymptomatic ICAS in a Chinese community-based study population free of stroke, transient ischemic attack and coronary heart disease using samples from the Asymptomatic Polyvascular Abnormalities Community (APAC) study.

## Results

### Prevalence of ICAS

Out of the study population of 5345 participants, 698 of them were diagnosed of ICAS based on the TCD results, representing a prevalence of 13.1%. Among them, 6.4% (342/5345) had middle cerebral artery stenosis, 4.3% (230/5345) had anterior cerebral artery stenosis, 1.1% (58/5345) had posterior cerebral artery stenosis, 1.2% (66/5345) had vertebral artery stenosis and 1.7% (89/5345) had basilar artery stenosis.

### Baseline characteristics

The baseline characteristics of all participants are presented in Table 1. As the number of each clinical TG level were in skewed distribution, medians were used for analysis, the median values were 93.0, 170.1, 270.7, respectively. There were significant differences for age, sex, current smoking status, hypertension, diabetes, body mass index (BMI), estimated glomerular filtration rate (e-GFR), TC, HDL-C and LDL-C (P < 0.05). Except for age and HDL-C whose absolute value decreased as the TG level increased, the rest of these variables shown the opposite tendency. Those with higher TG level were more likely to be men, smokers, suffering from hypertension and diabetes.

**Table 1:** Median baseline characteristics of participants according to triglyceride levels.

	Triglyceride levels			P value
	1: <1.7 mmol/L	2: 1.7–2.3 mmol/L	3: ≥2.3 mmol/L	
N	3656	735	954	
Age, years	55.7	54.6	53.5	<0.01
Male, %	59.0	60.7	64.0	0.02
Smoking, %	29.5	34.0	40.5	<0.01
Hypertension, %	43.4	54.7	59.5	<0.01
Diabetes, %	9.5	14.8	18.76	<0.01
BMI, kg/m^2^	24.3	25.9	26.4	<0.01
eGFR, ml/min·1.73 m^2^	90.2	93.8	95.1	<0.01
TC, mg/dl	188.9	204.4	214.5	<0.01
HDL-C, mg/dl	65.0	60.5	57.0	<0.01
LDL-C, mg/dl	99.9	107.8	103.5	<0.01

BMI: body mass index; eGFR: estimated glomerular filtration rate; TC: total cholesterol; HDL-C: high-density lipoprotein cholesterol; LDL-C: low-density lipoprotein cholesterol.

### Correlation between baseline FTG levels and the prevalence of asymptomatic ICAS

In univariate analysis, the association between FTG levels and asymptomatic ICAS didn’t reach statistical significance (OR: 0.99, 95% CI: 0.89–1.09; P = 0.79). In the multivariate analysis, ICAS were included as dependent variable and the presence of other parameters included as independent variables. After adjustment for age and sex, there was still no evidence shown any correlation between FTG levels and the presence of asymptomatic ICAS (OR: 1.05, 95% CI: 0.95–1.17). Moreover, the result was remained insignificance after the adjustment of age, sex, current smoking status, hypertension, diabetes, BMI, e-GFR, TC, HDL-C and LDL-C (OR: 0.95, 95% CI: 0.84–1.06), additional detailed information were displayed in Table 2.

**Table 2:** Odds ratios (OR) for ICAS according to baseline blood fasting triglyceride levels.

	Median	N (%)	Model1* (95% CI)	Model2^†^ (95% CI)
1	93.0	3656(68.4%)	1	1
2	170.1	735(13.8%)	1.12(0.88–1.42)	0.96(0.75–1.12)
3	270.7	954(17.8%)	1.09(0.87–1.35)	0.89(0.70–1.23)
P for trend			0.35	0.33
Continuous Scale			1.05(0.95–1.17)	0.95(0.84–1.06)

95% CI, 95% confidence interval.

*Model1: adjusted for age and sex. ^†^Model2: adjusted for age, sex, current smoking status, hypertension, diabetes, BMI, eGFR, TC, HDL-C and LDL-C.

Sex and other selected risk factors were stratified for further analyses of the association between FTG levels and the prevalence of asymptomatic ICAS, and there was no significant association in each subgroup (Table 3).

**Table 3:** Multivariate-adjusted odd ratios* (OR) for ICAS according to fasting triglyceride levels, stratified by sex and selected risk factors.

	Triglyceride levels			P for trend	Continuous Scale	P interaction
	1	2	3			
Sex						P = 0.05
Men	1	0.72(0.51–1.01)	0.90(0.67–1.21)	0.27	0.92(0.79–1.07)	
Women	1	1.54(1.07–2.21)	0.98(0.67–1.44)	0.65	1.04(0.87–1.25)	
Age						P = 0.40
<60	1	1.16(0.85–1.58)	0.88(0.66–1.19)	0.56	0.96(0.83–1.11)	
≥60	1	0.77(0.51–1.15)	1.01(0.69–1.49)	0.74	0.97(0.80–1.17)	
Hypertension						P = 0.32
No	1	1.22(0.81–1.85)	0.90(0.58–1.39)	0.87	0.98(0.80–1.21)	
Yes	1	0.85(0.63–1.15)	0.86(0.65–1.13)	0.22	0.92(0.80–1.05)	
Diabetes						P = 0.62
No	1	1.01(0.77–1.33)	0.91(0.70–1.19)	0.54	0.96(0.85–1.09)	
Yes	1	0.71(0.41–1.24)	0.74(0.46–1.19)	0.18	0.85(0.67–1.08)	
Smoking						P = 0.09
No	1	1.13(0.84–1.50)	0.98(0.74–1.30)	0.96	1.00(0.88–1.15)	
Yes	1	0.64(0.40–1.02)	0.72(0.48–1.08)	0.06	0.82(0.67–1.01)	

^*^Multivariate-adjusted odd ratios (OR): adjusted for age, sex, BMI, hypertension, diabetes, eGFR, HDL-C, LDL-C.

## Discussion

This study failed to show any significant relationship between FTG levels and the prevalence of asymptomatic ICAS. Several mechanisms can possibly explain the negative result.

First, increasing evidence show that it is TG-rich lipoproteins (TRLs), including chylomicrons, VLDL, and their remnants, rather than TG itself, contribute to the progression of atherosclerosis^17^. TRLs and their remnants are a cluster of particles which are heterogeneous in size, density as well as composition. There was a meaningful phenomenon that individuals with severe hypertriglyceridemia >100 mmol/L (88000 mg/dL) who suffered from chylomicronemia syndrome caused by lipoprotein deficiency was less likely to develop atherosclerosis than the mild-to-moderate hypertriglyceridemia, which could be explained by the fact that chylomicrons, the major part of TRLs in severe hypertriglyceridemia, were too large to enter the arterial endothelium^18^. While, the rest of TRLs are small enough to causing intimal cholesterol deposition directly. The TRLs also involved in a series of indirect pathways, including proinflammatory, proapoptotic, and procoagulant^19^, leading to atherosclerosis together. Furthermore, human genetic studies focusing on TRLs metabolism, supported its contribution to human ASCVD, including APOC3, APOA5, ANGPTL3 and many others^20–23^. Indicating that evaluating TG alone is limited, TG is merely a biomarker of TRLs and their remnants, the latter is atherogenic.

Our study discussing the FTG level, while people are in postprandial state for most of the day, leading to a hypothesis that postprandial TG (PTG) may play a more important role in the etiology of atherosclerosis than FTG. The Women’s Health Study, enrolled 20118 fasting and 6391 nonfasting participants, showed that only non-FTG levels were associated with incident cardiovascular events after adjustment for other risk factors, and the TG levels measured between 2 to 4 hours after a meal showed the strongest association^24^. Additionally, several other epidemiological studies have shown that the non-FTG levels is more closely associated with ASCVD than FTG^25–27^, which led to a change in the guideline. In the Endocrine Society Clinical Practice Guideline, it recommended the diagnose of hypertriglyceridemia on FTG, because the lack of a standard measurement on non-FTG levels^28^. The recently updated consensus statement by the European Atherosclerosis Society recommended that non-FTG can be used for risk evaluation and general screening^29^. And TG level has a high intra-individual variability which requires a more standardized method of measurement.

A majority of available literatures suggested that TG was a considerable risk factor for ASCVD, but when it comes to the relationship with possible ischemic stroke, an inconsistent result was obtained. A recent meta-analysis regarding to that, included 7 case-control studies revealed a significant odds ratio of 1.15 (95% CI, 1.08–1.21), but the significance was attributed to a single study only^30^, another systematic review also drew the similar conclusion^31^. The underlying mechanisms may attribute to the risk factors between them are different, the diameter of the vessel as coronary artery are larger than intracranial artery (ICA), as well as, ICA had greater antioxidant enzymes than other arteries of the same size^32^.

What’s more, a skewed distribution in TG levels can be seen in our study, the borderline-high TG group have a number of 735 (13.8%), the high TG group have a number of 954 (17.8%), the rest of the participants are all in the normal group, which may weaken the likelihood of positive result. Another limitation of our study is that when people with inadequate temporal window for TCD, we put them into the non-ICAS group. This may lead to an underestimation of ICAS prevalence. As a result of the observational design, residual confounding may be present because of remaining unknown, unmeasured, or inaccurately measured confounding factors. It is noteworthy that this is the first study investigating the association between FTG and the prevalence of ICAS. TG is a biomarker for atherosclerosis absolutely, but our study failed to show any expecting result for the reasons mentioned above.

In conclusion, FTG failed to show any statistical significance on ICAS presence in the APAC study, and the decades-long debate continues. But the TG level actually plays an important role in the progression of atherosclerosis as a biomarker, additional lifestyle intervention together with TG-lowering agents can reduce the residual risk. Further epidemiological studies are needed to investigate the relationship between them.

## Subjects and Methods

### Study Population

The APAC study is a community-based, prospective, long-term follow-up observational study targeting on the epidemiology of asymptomatic polyvascular abnormalities in Chinese adults. The study was sub-population of the Kailuan study which described previously^33^. A total of 5852 people stratified randomly by age and sex agreed to join the APAC study, and 5816 participants eventually completed the baseline data collection from June 2010 to June 2011. Among these 5816 participants, 376 did not meet the inclusion criteria: (1) aged 40 years or older; and (2) no history of stroke, transient ischemic attack, and coronary heart disease at baseline. Besides, 90 subjects with incomplete information on TG data, vascular examination results or under lipid-lowering treatment were further excluded. Finally, 5345 participants were available in this study, where 3212 participants are men.

The study was performed according to the guidelines from the Helsinki Declaration and was approved by the Ethics Committees of the Kailuan General Hospital and the Beijing Tiantan Hospital. Written informed consent was obtained from all participants. Subjects were also informed of abnormal findings and recommended treatment.

### Measurement of Indicators

A questionnaire was used to obtain baseline information by trained investigators on enrolled participants, containing age, sex, smoking status, past medical history such as hypertension, diabetes, hyperlipidemia, and medications prescribed by physicians. The baseline data were further categorized by different parameters. Age was classified into “<60 years” and “≥60 years”. Smoking status was classified into “non-smoking” and “smoking” (current smoker who smokes at least one cigarette per day) by self-reported information.

### Definition of hypertension, diabetes and hyperlipidemia

Hypertension was diagnosed if the subject had a history of hypertension, or a systolic blood pressure (SBP) ≥140 mmHg, or a diastolic blood pressure ≥90 mmHg, or currently taking antihypertensive medication. Diabetes mellitus was diagnosed if the subjects had a history of diabetes mellitus, or currently undergoing treatment with insulin or oral hypoglycemic, or the fasting blood glucose level was ≥7.0 mmol/L. Hyperlipidemia was diagnosed if the subject had a history of hyperlipidemia, or currently using cholesterol lowering agents, or the TC level ≥ 5.17 mmol/L or TG ≥ 1.7 mmol/L.

### Physical examination

Body weight, height was measured and then BMI was calculated as body weight (kg) divided by the square of height (m^2^). For blood pressure, the participants should sit quietly for 5 minutes before the measurement, an average of two readings was used for the current study. If the two measurements differed by more than 5 mmHg, a third reading was taken, and the average of the three readings was used.

### Biochemical Index

Blood samples were obtained from the antecubital vein after an overnight fasting in EDTA tubes and analyzed within 4 h of preparation using an auto-analyzer (Hitachi 747; Hitachi, Tokyo, Japan) at the central laboratory of the Kailuan hospital. Estimated GFR (eGFR) was calculated by using a modified 4-variable Chronic Kidney Disease Epidemiology Collaboration (CKD-EPI) formula with an adjusted coefficient of 1.1 for the Chinese population to estimate eGFR^34^. TC was measured using the endpoint test method. HDL-C and LDL-C levels were measured using a direct test method and TG were measured using the GPO method (inter-assay coefficient of variation: <10%; Mind Bioengineering Co. Ltd, Shanghai, China).

The following is the criteria for clinical diagnosis of elevated triglyceride levels under fasting conditions according to the Endocrine Society 2010 Guideline. Normal TG: <1.7 mmol/L, borderline-high TG: 1.7–2.3 mmol/L, high TG: 2.3–11.1 mmol/L, very high TG: 11.2–22.4 mmol/L and very severe ≥22.4 mmol/L^28^ (to convert triglyceride values to mg/dL multiply by 88.6). Accordingly, we classify the last 3 group together as ≥2.3 mmol/L in consideration of the limited number of participants.

### Trans-cranial Doppler Ultrasonography (TCD) Examination

TCD, as a non-invasive, reliable and available method to diagnose ICAS^35^, was performed by two experienced neurologists using portable devices (EME Companion, Nicolet, Madison, WI, USA). The two neurologists were blinded to the baseline information of the participants. ICAS was defined according to the peak flow velocity criteria that was published and validated against MR angiography (MRA) and clinical outcomes^36^. In short, ICAS was defined by a peak systolic flowing velocity (Vp) >140 cm/s for the middle cerebral artery; >120 cm/s for the anterior cerebral artery and internal carotid siphon; and >100 cm/s for the posterior cerebral artery and vertebral-basilar. In addition to the above mentioned criteria, the patients’ age, the presence of turbulence sound or disturbance in echo frequency, and whether the abnormal velocity was segmental were also taken into account for ICAS diagnosis. Subjects without a good temporal window were considered without stenosis. ICAS was diagnosed if at least one of the studied arteries showed evidence of stenosis or occlusion.

### Statistical analysis

We classified the participants into three groups according to clinical diagnosis of elevated triglyceride levels. All statistical analyses were performed using SAS software, version 9.1 (SAS Institute, Cary, North Carolina, USA). Continuous variables were compared using analysis of variance (ANOVA) and categorical variables were compared using chi-square tests. The age- and sex-adjusted or multivariate-adjusted odd ratios (OR) and 95% confidence interval (CI) were calculated using logistic regression model on the relationship between FTGlevels and ICAS. The multivariate-adjusted model were further adjusted for age, sex, current smoking status, hypertension, diabetes, BMI, e-GFR, TC, HDL-C and LDL-C levels. A trend test was applied to examine whether there was a dose-dependent relationship between the three clinical levels of FTG and ICAS prevalence. Categorical data were treated as continuous data using the median level of FTG levels in each group. Additionally, sex and other potential indicators were also evaluated in subgroups, to assess if there was any significant interaction between these variables and the relationship between FTG levels and ICAS prevalence. All statistical analyses were two-tailed, and a P value less than 0.05 was considered statistically significant.
