# Author Correction: Association between fasting Triglyceride levels and the Prevalence of Asymptomatic Intracranial Arterial Stenosis in a Chinese Community-based Study

**DOI:** 10.1038/s41598-019-42890-8

**Published:** 2019-05-17

**Authors:** Jianwei Wu, Yu Wang, Anxin Wang, Jian Xie, Xingquan Zhao

**Affiliations:** 1 Department of Neurology, Beijing Tiantan Hospital, Capital Medical University, Beijing, 100050 China; 2 China National Clinical Research Center for Neurological Diseases, Beijing, 100050 China; 3 Center of Stroke, Beijing Institute for Brain Disorders, Beijing, 100050 China; 4 Center of Brain Tumor, Beijing Institute for Brain Disorders, Beijing, 100050 China; 5 Beijing Key Laboratory of Translational Medicine for Cerebrovascular Disease, Beijing, 100050 China; 6 Beijing Key Laboratory of Brain Tumor, Beijing, 100050 China; 7 Department of Epidemiology and Health Statistics, School of Public Health, Capital Medical University, Beijing, 100050 China; 8 Department of Neurosurgery, Beijing Tiantan Hospital, Capital Medical University, Beijing, 100050 China

Correction to: *Scientific Reports* 10.1038/s41598-018-24157-w, published online 10 April 2018

In the original version of this Article, Jian Xie was incorrectly affiliated with ‘Department of Neurology, Beijing Tiantan Hospital, Capital Medical University, Beijing, 100050, China’. The correct affiliation is listed below.

Department of Neurosurgery, Beijing Tiantan Hospital, Capital Medical University, Beijing, 100050 China

This error has now been corrected in the PDF and HTML versions of the Article.

